# Retroperitoneal Myolipoma with Hip Invasion: A Case Report

**DOI:** 10.3390/reports9010077

**Published:** 2026-03-06

**Authors:** Bassel El Osta, Luigi Di Lorenzo, Andrea Vescio, Laura Campanacci, Hassan Zmerly

**Affiliations:** 1 Faculty of Medecine, Balamand University, Balamand 55251, Lebanon; dr.belosta@gmail.com; 2 Department of Life Sciences, Health, and Health Professions, Link Campus University, 00165 Rome, Italy; l.dilorenzo@unilink.it (L.D.L.); a.vescio@unilink.it (A.V.); 3 Third Orthopedic and Traumatologic Clinic Prevalently Oncologic, IRCCS Istituto Ortopedico Rizzoli, 40136 Bologna, Italy; laura.campanacci@ior.it; 4 Department of Orthopedics, Villa Erbosa Hospital, Gruppo San Donato, 40129 Bologna, Italy

**Keywords:** myolipoma, neoplasm, soft tissue tumors, pelvis, retroperitoneal, hip, excision

## Abstract

**Background and Clinical Significance**: Myolipoma is a rare benign tumor, typically found in the retroperitoneum and characterized by a combination of mature adipocytes and well-differentiated smooth muscle cells. Myoplipomas usually present a delay in diagnosis due to the painless and slow-growing clinical behavior; therefore, the lesion can reach a large dimension with challenging treatment. **Case Presentation**: We present the case of a retroperitoneal myolipoma infiltrating the left hip of an 11-year-old male. It was suspected based on magnetic resonance imaging. The patient has been successfully treated with surgical excision without complications. Histological examination revealed mature adipose tissue infiltrating smooth muscle cells. The muscle fibers appeared normal, while the dense connective tissue was infiltrated by clusters of mature lymphocytes. **Conclusions**: Although myolipoma is extremely rare in male children and has never been reported to infiltrate the hip, it should be considered in the differential diagnosis of fat-containing retroperitoneal masses.

## 1. Introduction and Clinical Significance

Soft-tissue lipoma is a rare benign mesenchymal neoplasm that typically presents as a painless, slow-growing mass. It most commonly affects adult males between the fifth and seventh decades of life. According to its anatomical location, lipomas are classified as superficial (subcutaneous) or deep (muscular). Deep lipomas are further subdivided into intermuscular and periosteal types; however, they may also infiltrate skeletal muscle (intramuscular) or bone (intraosseous) [1,2]. Lipomas may occur in association with other mesenchymal components, resulting in histological variants such as myolipoma. Myolipomas, infiltrating smooth muscle, were first described by Meis and Enzinger in 1991. They are mainly composed of mature adipocytes and well-differentiated smooth muscle cells. Myolipomas have been most frequently reported in the retroperitoneum. Additional reported sites include the subcutaneous tissue, orbital region, pericardium, rectus sheath of the anterior abdominal wall, and the abdominal cavity, where they may be attached to the abdominal wall [2,3,4]. We report the clinical, radiological, and histological features of a rare case of an abdominal myolipoma infiltrating the left hip, in which a delayed diagnosis resulted in a challenging therapeutic management.

## 2. Case Presentation

### 2.1. History

We present the case of an 11-year-old male patient who was evaluated at the outpatient clinic of our hospital with a 3-month history of left hip pain radiating to the left thigh, particularly when asleep. The patient was born through vaginal delivery with no complications and is healthy with no previous medical history. Upon physical examination, there was tenderness over the left hip with a decreased range of motion, particularly in adduction. No swelling was detected.

### 2.2. Diagnostic Approach

Laboratory examinations revealed an elevated sedimentation rate (29 mm/1 h, normal value below 10 mm/1 h), insufficient 25-OH vitamin D3 (22.8 ng/mL, value of moderate deficiency 12.5–29 ng/mL), and normal phosphatase alkaline (190 U/L). The laboratory results determined that the patient was negative for Anti-CCP (Anti-Cyclic Citrullinated Peptide).

An X-ray of the hip and left femur was taken and showed no visible bone or joint lesion. The patient was treated by a general orthopedic surgeon and was given NSAIDs (Ibuprofen) and Paracetamol for symptom relief.

Due to the persistence of symptoms, an MRI of both hips was performed, and it showed a moderate amount of left joint effusion associated with significant synovitis and adjacent soft tissue edema involving the obturator muscle with resultant mild dislocation of the femur, remaining partially covered by the acetabular edge. The patient was treated conservatively with rest, physiotherapy, and the use of crutches and NSAIDs for symptom relief.

**Figure 1:**
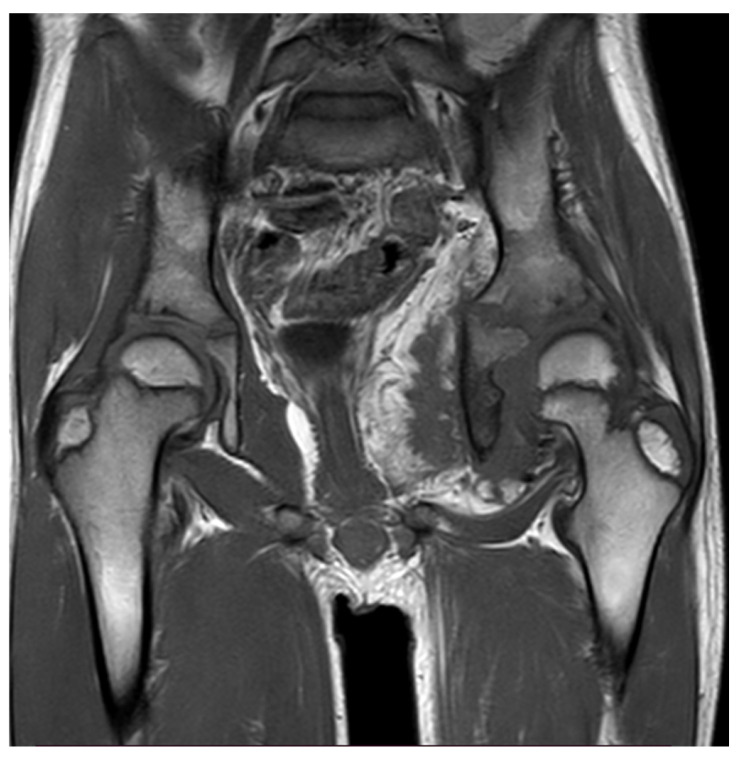
The MRI showing large heterogeneous soft-tissue mass in the abdominal cavity, with extension to the hip joint.

Thirteen months later, the patient underwent a new pelvis MRI examination due to the symptoms worsening and a significant decrease in the range of motion of the left hip. MRI revealed a large heterogeneous soft-tissue mass in the abdominal cavity, with extension to the hip joint, presenting both fat and muscle signal intensity with heterogeneous enhancement post gadolinium, and associated with extensive peri-osseous fatty infiltration, a reactive soft-tissue component, and advanced severe atrophy of the left obturator muscles (Figure 1). These findings were not observed in the previous MRI.

At this stage, an incisional biopsy was performed, which revealed no pathological tissue.

Due to the persistence of symptoms, the patient underwent a follow-up MRI six months later, which showed no significant changes compared with the previous images. Therefore, the orthopedic surgeon decided, together with the urology consultant, to operate on the hip as well as the abdominal area.

### 2.3. Surgical Treatment

The surgical approach employed was an extended Smith–Petersen approach, continued medially to the midline through a horizontal incision. Intraoperatively, guided by preoperative MRI findings, the surgeon traced the diseased tissue and identified its attachment to the lateral internal pelvic wall, with extension through the ischium toward the acetabulum.

The mass presented as a smooth capsule. Once the neoplasm was dissected, it was completely released from the acetabulum, resulting in restoration of hip mobility.

### 2.4. Pathological Finding

**Figure 2:**
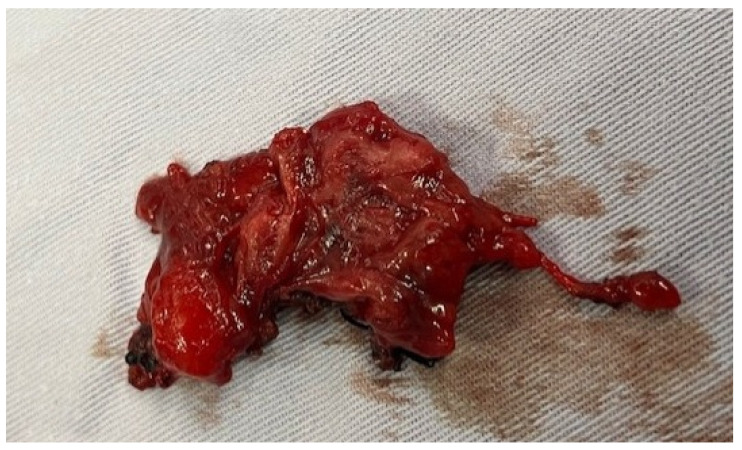
Macroscopic aspect of the excised mass.

On gross examination, the mass had a solid, rubbery consistency, was well circumscribed, and measured 23 cm in greatest dimension. (Figure 2).

Histological examination of multiple specimens obtained from four different sites (soft tissue, bone, tumor, and hip) revealed mature adipose tissue infiltrating smooth muscle cells. The muscle fibers appeared normal, while the dense connective tissue showed infiltration by clusters of mature lymphocytes. No features of malignancy were identified. These findings supported the diagnosis of a myolipoma infiltrating the hip.

### 2.5. Follow-Up

At 1, 6, 12 and 24 months follow-up, the patient was free of symptoms. Physical and ultrasound examinations showed a favorable outcome without evidence of disease.

## 3. Discussion

Lipomas are the most common benign mesenchymal neoplasm in humans. They usually grow in male adults aged between 50 and 70. Lipomas can occur in combination with other mesenchymal elements, giving rise to variants including myolipoma that infiltrate smooth muscle [5,6,7].

Unlike typical lipomas, myolipomas have a slightly higher tendency to occur in women during their fifth and sixth decades of life. As for their localization, the retroperitoneum is the most common location for their growth, followed by less common localizations like sub-cutaneous tissue, the orbital region, the pericardium, the rectus sheath of the anterior abdominal wall and erector spinae [7].

In contrast, our patient is a young male with a retroperitoneal myolipoma infiltrating the left hip, a location that, to the best of our knowledge, has yet to be reported in the literature.

The histological aspect of these tumors consists of variable amounts of benign smooth muscle fibers and mature adipose tissue with no lipoblasts, floret-like giant cells or zones of atypia. Cellular areas composed of bundles of spindle-shaped eosinophilic cells and reminiscent of smooth muscle are dispersed within the mature adipose tissue cells. Myolipoma may be misdiagnosed as other adipocytic or mesenchymal tumors, including well-differentiated liposarcoma, spindle cell lipoma, angiomyolipoma, leiomyoma with fatty degeneration, lipoleiomyosarcoma, and leiomyosarcoma [3,7]. Myolipomas are often confused with well-differentiated liposarcoma due to their similar fat content. Histologically, liposarcomas contain lipoblasts or floret-like giant cells, are encapsulated and show a fatty mass with poorly defined internal areas of non-adipose tissue [2]. Pathogenesis of myolipoma remains unclear. There are two main theories, namely adipose metaplasia and multipotential Mullerian cell origin [6,7,8,9].

Imaging is considered the gold standard for the diagnosis of myolipoma. Ultrasound may suggest the presence of a myolipoma, as the lipomatous regions of the tumor appear hyperechogenic; however, it cannot provide a definitive diagnosis. On CT and MRI, the lipomatous areas demonstrate typical fat characteristics. In contrast, the non-lipomatous regions display nonspecific solid features, with soft-tissue attenuation on CT, intermediate signal intensity on T1-weighted MRI, and intermediate to high signal intensity on T2-weighted MRI [5]. The MRI conducted on our patient showed extensive peri-osseous fatty infiltration by soft tissue reaction, which correlates with the fact that MRI shows the features of fat tissue in lipomatous components of myolipoma.

A preoperative biopsy is necessary to confirm the diagnosis, particularly in lesions larger than 5 cm. In our case, the histological findings from the preoperative biopsy revealed no pathological tissue, likely because the specimen was obtained from a non-representative area of the lesion. Therefore, a follow-up MRI was obtained 6 months after the previous examination, which showed no change in the fatty lesion, and surgical excision was subsequently undertaken due to the persistence of symptoms.

Surgical excision is the optimal treatment of myolipoma [2,10]. No cases of local recurrence, metastatic disease or malignant transformation have been reported in the literature. In the follow-up of soft tissue tumors, clinical evaluation, ultrasound and MRI represent the examination of choice. However, in our case, we preferred to perform only clinical and ultrasound examination because the patient was an 11-year-old child who was uncomfortable undergoing an MRI. Moreover, the lesion was benign, and there were no clinical signs suggestive of recurrence.

This case is reported due to its extreme rarity that made the diagnosis more difficult. To the best of our knowledge, this is the first case of a retroperitoneal myolipoma infiltrating the hip in a child.

## 4. Conclusions

Myolipoma is a rare tumor that should be considered in the differential diagnosis of retroperitoneal masses. MRI is highly useful in the evaluation of these tumors and can raise suspicion for a myolipoma, guiding further diagnostic assessment. Histological examination is mandatory to confirm the histopathologic aspect, showing a common adipose tissue infiltrating a skeletal striated muscle.

In the rare event of retroperitoneal myolipoma infiltrating the hip joint, surgical excision is the treatment of choice that can lead to complete recovery.

## Data Availability

The original data presented in this study are available on reasonable request from the corresponding author. The data are not publicly available due to privacy concerns.

## Abbreviations

The following abbreviations are used in this manuscript:

NSAIDs	Nonsteroidal anti-inflammatory drugs
MRI	Magnetic Resonance Imaging
